# Defective Chylomicron Synthesis as a Cause of Delayed Particle Clearance in Diabetes?

**DOI:** 10.1080/15604280214277

**Published:** 2002

**Authors:** Catherine Phillips, Claire Madigan, Daphne Owens, Patrick Collins, Gerald H. Tomkin

**Affiliations:** 1 Department of Clinical Medicine Trinity College Dublin Ireland; 2 Department of Biochemistry The Royal College of Surgeons in Ireland Dublin Ireland; 3 Department of Endocrinology and Diabetes Adelaide/Meath Hospital Dublin Ireland; 4 1, Fitzwilliam Square Dublin 2 Ireland

## Abstract

Chylomicron metabolism is abnormal in diabetes and the chylomicron particle may play a very important role in atherosclerosis. The aim of this study was to examine the effect of diabetes on the metabolism of chylomicrons in cholesterol-fed alloxan diabetic and nondiabetic rabbits. Five diabetic rabbits and 5 control rabbits were given [^14^C]linoleic acid and [^3^H]cholesterol by gavage. Lymph was collected following cannulation of the lymph duct and radiolabelled chylomicrons were isolated by ultracentrifugation. The chylomicrons from each animal were injected into paired control and diabetic recipients. Lymph apolipoprotein (apo) B48, apo B100, and apo E were measured using sodium dodecyl sulfate–polyacrylamide gradient gel electrophoresis. Mean blood sugar of the diabetic donors and diabetic recipients were 19.7 ± 2.3 and 17.2 ± 3.2 mmol/L. Diabetic rabbits had significantly raised plasma triglyceride (10.8 ± 13.9 versus 0.8 ± 0.5 mmol/L, *P* < 0.02). There was a large increase in apo B48 in lymph chylomicrons in the diabetic donor animals (0.19 ± 0.10 versus 0.04 ± 0.02 mg/h, *P* < 0.01) and apo B100 (0.22 ± 0.15 versus 0.07 ± 0.07 mg/h, *P* < 0.05) and a reduction in apo E on the lymph chylomicron particle (0.27 ± 0.01 versus 0.62 ± 0.07 mg/mg apo B, *P* < 0.001). Diabetic recipients cleared both control and diabetic chylomicron triglyceride significantly more slowly than control recipients (*P* < 0.05). Clearance of control chylomicron cholesterol was delayed when injected into diabetic recipients compared to when these chylomicrons were injected into control recipients (*P* < 0.005). Clearance of diabetic chylomicron cholesterol was significantly slower when injected into control animals compared to control chylomicron injected into control animals (*P* < 0.02). In this animal model of atherosclerosis, we have demonstrated that diabetes leads to the production of an increased number of lipid and apo E–deficient chylomicron particles. Chylomicron particles from the control animals were cleared more slowly by the diabetic recipient (both triglyceride and cholesterol). The chylomicron particles obtained from the diabetic animals were cleared even more slowly when injected into the diabetic recipient. Although there was an initial delay in clearance of chylomicron triglyceride from the diabetic particle when injected into the control animals, the clearance over the first 15 minutes was not significantly different when compared to the control chylomicron injected into the control animal. On the other hand, the cholesterol clearance was significantly delayed. Thus, diabetes resulted in the production of an increased number of lipid- and apo E–deficient chylomicron particles. These alterations account, in part, for the delay in clearance of these particles.


*Int. Jnl. Experimental Diab. Res.*, 3:171–178, 2002
Copyright © 2002 Taylor & Francis
1560-4284/02 $12.00 + .00
DOI: 10.1080/15604280290013874

Taylor & Francis healthsciences

### Defective Chylomicron Synthesis as a Cause of Delayed Particle Clearance in Diabetes?

**Catherine Phillips,[1] Claire Madigan,[1] Daphne Owens,[1] Patrick Collins,[2] and Gerald H. Tomkin[1,3]**

[1]*Department of Clinical Medicine, Trinity College, Dublin, Ireland*
[2]*Department of Biochemistry, The Royal College of Surgeons in Ireland, Dublin, Ireland*
[3]*Department of Endocrinology and Diabetes, Adelaide/Meath Hospital, Dublin, Ireland*

**Chylomicron metabolism is abnormal in diabetes and the chylomicron particle may play a very important role in atherosclerosis. The aim of this study was to examine the effect of diabetes on the metabolism of chylomicrons in cholesterol-fed alloxan diabetic and nondiabetic rabbits. Five diabetic rabbits and 5 control rabbits were given [$^{14}$C]linoleic acid and [$^{3}$H]cholesterol by gavage. Lymph was collected following cannulation of the lymph duct and radiolabelled chylomicrons were isolated by ultracentrifugation. The chylomicrons from each animal were injected into paired control and diabetic recipients. Lymph apolipoprotein (apo) B48, apo B100, and apo E were measured using sodium dodecyl sulfate–polyacrylamide gradient gel electrophoresis. Mean blood sugar of the diabetic donors and diabetic recipients were 19.7 ± 2.3 and 17.2 ± 3.2 mmol/L. Diabetic rabbits had significantly raised plasma triglyceride (10.8 ± 13.9 versus 0.8 ± 0.5 mmol/L, $P < 0.02$). There was a large increase in apo B48 in lymph chylomicrons in the diabetic donor animals (0.19 ± 0.10 versus 0.04 ± 0.02 mg/h, $P < 0.01$) and apo B100 (0.22 ± 0.15 versus 0.07 ± 0.07 mg/h, $P < 0.05$) and a reduction in apo E on the lymph chylomicron particle (0.27 ± 0.01 versus 0.62 ± 0.07 mg/mg apo B, $P < 0.001$). Diabetic recipients cleared both control and diabetic chylomicron triglyceride significantly more slowly than control recipients ($P < 0.05$). Clearance of control chylomicron cholesterol was delayed when injected into diabetic recipients compared to when these chylomicrons were injected into control recipients ($P < 0.005$). Clearance of diabetic chylomicron cholesterol was significantly slower when injected into control animals compared to control chylomicron injected into control animals ($P < 0.02$). In this animal model of atherosclerosis, we have demonstrated that diabetes leads to the production of an increased number of lipid and apo E–deficient chylomicron particles. Chylomicron particles from the control animals were cleared more slowly by the diabetic recipient (both triglyceride and cholesterol). The chylomicron particles obtained from the diabetic animals were cleared even more slowly when injected into the diabetic recipient. Although there was an initial delay in clearance of chylomicron triglyceride from the diabetic particle when injected into the control animals, the clearance over the first 15 minutes was not significantly different when compared to the control chylomicron injected into the control animal. On the other hand, the cholesterol clearance was significantly delayed. Thus, diabetes resulted in the production of an increased number of lipid- and apo E–deficient chylomicron particles. These alterations account, in part, for the delay in clearance of these particles.**

**Keywords** Apolipoprotein B48; Apolipoprotein B100; Apolipoprotein E; Cholesterol Turnover; Chylomicron Turnover; Triglyceride Turnover; Type 2 Diabetes

Postprandial lipoprotein metabolism is considerably altered in diabetes [1, 2], a condition that is associated with an up to 4-fold increase in atherosclerosis. Postprandial hyperglycemia has been confirmed as a risk factor for mortality [3] and new treatments with both quick-acting synthetic insulin and short-acting oral hypoglycemic agents, which significantly reduce postprandial hyperglycemia, are promoted as being beneficial [4]. So far, there has been little interest in the effect of improved glycemic control on the postprandial dyslipidemia of diabetes. This may be due to our poor understanding of the metabolism of postprandial lipoprotein particles. Our previous studies on postprandial lipoprotein metabolism in diabetes have demonstrated abnormalities in the intestinally derived apolipoprotein

Received 20 November 2001; accepted 24 January 2002.
The study was funded by grants from the Diabetes Federation of Ireland and The Health Research Board of Ireland. The authors are grateful to Ms. Philipa Marks, Trinity College, Dublin, for carrying out the surgical procedures and to Dr. Ronan Conroy, Royal College of Surgeons in Ireland, for statistical advice.
Address correspondence to Professor G. H. Tomkin, 1, Fitzwilliam Square, Dublin 2, Ireland. E-mail: gtomkin@rcsi.ie

(apo) B48–containing particles and also in the hepatically derived apo B100–containing particles [5–7]. In diabetic patients, we have demonstrated an increase in the number of particles and an increase in both the cholesterol and triglyceride contents of each particle [8]. Studies in rats, a model that does not develop atherosclerosis, have been conflicting. Levy and coworkers [9] have shown that the triglyceride from a chylomicron particle obtained from a diabetic rat, when injected into a nondiabetic rat, is cleared more slowly. On the other hand, Feingold and colleagues [10] showed that a [$^{14}$C]cholesterol-labeled diabetic chylomicron, when injected into a nondiabetic rat, was cleared at the same rate as a nondiabetic chylomicron. Apo E, synthesised both by the liver and peripheral tissues including the intestine, plays an important role in mediating the hepatic recognition and uptake of the chylomicron. It serves as a ligand for the low-density lipoprotein (LDL) B/E receptor, the LDL receptor–related protein (LRP), the lypolysis-stimulated receptor (LSR), and for cell surface heparin sulphate proteoglycans [11–13]. A reduction in apo E mRNA expression was demonstrated in cholesterol-fed diabetic rabbits [14], suggesting that insulin deficiency downregulates apo E mRNA expression. Overexpression of apolipoprotein E in transgenic mice has been shown to prevent the development of diabetic hyperlipidemia [15]. Cannulation of the lymphatic duct allows examination of lipoprotein particles prior to "first pass" alteration, which may be particularly important in the understanding of the chylomicron, because it is so rapidly cleared by the liver. The present study was undertaken to examine the impact of diabetes on metabolism of the intestinally derived chylomicron particle. We used the cholesterol-fed alloxan diabetic and nondiabetic rabbit as an animal model of diabetes and atherosclerosis [16].

### MATERIALS AND METHODS

### Animals

Male New Zealand white rabbits (Harlan, UK) (n = 30) were housed individually in a reverse light cycle (1 AM to 1 PM light, 1 PM to 1 AM dark). Rabbits were acclimatized for 1 week, with free access to standard chow and water, and were then changed to a 0.5% cholesterol diet (Special Diets Service, UK) ad libutim for 6 weeks. Atherosclerosis is easily induced by feeding rabbits cholesterol [16]. We used an atherosclerotic model because we were particularly concerned about why diabetes accelerates atherosclerosis. Animals were housed under license from the Department of Health and experiments were carried out according to Irish law as administered by the Department of Health.

At the beginning of the 5th week, diabetes was induced by intravenous infusion of a 10% (*w*/*v*) solution of alloxan monohydrate (150 mg/kg) in physiological saline through a catheter inserted via a marginal ear vein. To counteract hypoglycemia, caused by insulin release from necrotic beta cells in the pancreas, the rabbits were provided with a 20% (*w*/*v*) solution of glucose for the first 24 hours. Blood glucose was determined throughout this period and when animals became hypoglycemic, a 50% (*w*/*v*) glucose solution was given intragastrically. Diabetes was confirmed 48 hours later with blood glucose >22 mmol/L, as determined by glucotrend (Boehringer Mannheim, Mannheim, Germany) strip. Urine ketones were monitored with ketostix and rabbits were not ketotic. Blood glucose and food intake of each rabbit was monitored daily. Diabetic rabbits (n = 15) were kept in moderate control by daily subcutaneous injection of insulin (Ultratard, Novo Nordisk). Animals were diabetic for at least 8 days prior to experiment.

### Radiolabeling of Chylomicrons and Lymph Duct Cannulation

Diabetic (n = 5) and control (n = 5) rabbits were given, by gavage, 20 ml of an emulsion containing sunflower oil (75% *v*/*v*), $H_2O$ (25% *v*/*v*), phosphotidylcholine (5% *w*/*v*), and 100 $\mu$Ci [$^3$H]cholesterol and 50 $\mu$Ci [$^{14}$C]linoleic acid. Rabbits were returned to their cages with access to water. Five hours after gavage, rabbits were anesthetized by intramuscular injection of Hypnorm (0.3 ml/kg), followed by intravenous injection of Hypnovel (2 mg/kg). Rabbits were intubated and attached to a Penlon Nuffield anesthesia machine and administered oxygen and ethrane. A laparotomy was performed and the left kidney tied off and removed. The mesenteric lymph duct, which lies ventrally above the abdominal aorta, was tied off and cannulated by inserting a 2-mm portex tube. Tubing was exteriorized through a stab wound in the left side and positioned such that regular lymph flow occurred. Rabbits were given 50-ml/h warm saline intravenously. Lymph was collected for 4 hours into tubes containing EDTA (1 mg/ml) and antiproteases were added to prevent oxidation and degradation of the lipoproteins. At the end of the experiment, blood was taken for determination of plasma lipoproteins and apoproteins. Rabbits were sacrificed by an overdose of euthane.

### Preparation of Lymph and Plasma Chylomicrons

Lymph and plasma were overlaid with a 1.006-g/mL density solution and centrifuged at 20,000 rpm at 10°C for 30 minutes in a Beckman L7-55 ultracentrifuge using a fixed angle rotor. Chylomicrons were carefully removed from the top of the tube with a stretched pasteur pipette. Lymph chylomicrons (20 $\mu$l) were added to 4 ml of scintillation fluid and counted in a 1214 Rack Beta scintillation counter. Lymph and plasma chylomicrons were kept at 4°C and used for the turnover studies within 24 hours.

### Chylomicron Clearance

The clearance study was designed so that lymph chylomicrons from control rabbits were injected into paired control (n = 5) and diabetic (n = 5) rabbits and chylomicrons from diabetic rabbits were injected into paired control (n = 5) and diabetic rabbits (n = 5). Radiolabeled lymph chylomicrons (50 mg chylomicron triglyceride, specific activity 10,000 cpm/ mg lipid, approximately) were injected into a marginal ear vein of a conscious recipient rabbit. Blood was sampled from the opposite ear artery before and after injection, at regular intervals for up to 40 minutes. Blood was centrifuged at 2000 rpm for 10 minutes at 4°C to obtain plasma. Radioactivity in 250-$\mu$l samples of plasma was determined by liquid scintillation counting using a dual-label mode. In order to calculate the percentage of radioactivity in plasma after injection, total rabbit plasma volume was assumed to be 3.4% of rabbit body weight. Area under the curve from 0 to 15 minutes (area from 0 to 100 defined by the curve during the 15-minute period) for [$^3$H]cholesterol and [$^{14}$C]linoleic acid clearance was calculated.

### Lymph and Plasma Lipids

Plasma, lymph, and lipoprotein cholesterol were measured by enzymatic colorimetric methods, using commercially available kits (Boehringer Mannheim). Plasma, lymph, and lipoprotein triglycerides and phospholipids were measured with kits from BioMerieux (Charbonnieres les Bains, France). Plasma high-density liproprotein (HDL) was measured by using a HDL-C reagent (Boehringer Mannheim). Protein was estimated by a modification of the Lowry method [17].

### Quantification of apo B48, apo B100, and apo E

Rabbit lymph and plasma chylomicron apo B48, apo B100, and apo E were measured using a modification of the method described previously for human plasma apo B48 and apo B100 [8, 18]. Samples were partially delipidated by mixing with diethyl ether (1:6 *v/v*), centrifuging at 14,000 rpm for 15 minutes at room temperature, and removing the organic phase. The protein was dried down and redissolved in 25 $\mu$l denaturing buffer (2.0% [*v/v*] $\beta$-mercaptoethanol, 4.0% sodium dodecyl sulfate [SDS], 0.01% *v/v* bromophenol blue, 0.1 mol/l Tris-HCl, pH 6.8, 20% [*v/v*] glycerol), heated for 4 minutes at 96°C, and loaded on a 4% to 15% polyacrylamide gel (BioRad, Herculas, CA, USA). Electrophoresis was for 1 hour at 60 mA constant current in 0.019 mol/l Tris, 0.192 mol/l glycine. Gels were stained for 1 hour with Coomassie Brilliant Blue (0.1% in methanol:acetic acid:water 4:1:5) and destained with several changes of the same solvent (Figure 1). Because the chromogenicity of apo B48 has been shown to be similar to that of apo B100, and the chromogenicity of apo E has been reported to be twice that of apo

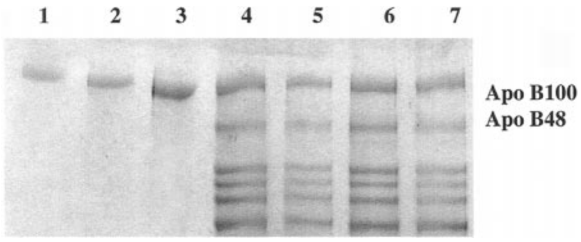

**FIGURE 1**
Typical SDS-PAGE gel of rabbit lymph chylomicron showing apo B48 and apo B100 separation. Lanes 1 to 3: apo B100 standards; lanes 4 to 7: lymph chylomicrons from 4 different rabbits showing apo B48 and apo B100 bands.

B100 [19], a protein standard was prepared from LDL (density 1.025 to 1.063 g/ml) of a single individual, was stored at −20°C, and used throughout the study for quantification of apo B48, apo B100, and apo E. Staining was linear within the range 0.1 to 2 $\mu$g of protein. Apo B100 protein standards (0.2, 0.6, 1.2, and 2 $\mu$g) were applied to all gels. The bands were quantified by densitometry using Vilber Lourmat equipment (Vilber Lourmat Biotechnology, Marne de Vallee, France). Video images of the gels were generated and imported into Bio1D v6.32 software for analysis (Figure 1). Density values were assigned to the apo B100 bands of the human LDL and a standard curve constructed. The values were recalculated by linear regression and curves with a correlation coefficient >.95 were accepted. The concentrations of apo B48, apo B100, and apo E were then determined from this standard. The intra- and interassay variations (n = 6) for apo B48 were 3.4% and 6.1%, for apo B100 were 5.9% and 8.3%, and for apo E were 1.8% and 3.6%.

### Statistical Analysis

Statistical analysis was performed using the Student *t* test (paired and unpaired). Area under the curve (AUC) measurements were obtained and correlation coefficients were determined by linear-regression analysis using Graphpad Prism 2 for Macintosh (Graphpad Software, San Deigo, CA USA). Inter- and intraassay variation were expressed as standard deviation/ mean ×100. Results were expressed as mean ± standard deviation. A $P$ value of $<0.05$ was regarded as statistically significant.

### RESULTS

Animal characteristics are shown in Table 1. The diabetic rabbits were slightly but not significantly lighter on the day of the experiment. There was no significant difference in the degree of diabetes between the diabetic donor and recipient rabbits on the day of the experiment and no difference in average daily insulin requirements. Examination of the plasma lipids revealed the expected increase in plasma triglyceride and decrease in plasma HDL in the diabetic rabbits. Total cholesterol was higher in the

**TABLE 1**
Characteristics and plasma lipids in diabetic and control rabbit after 6 weeks of a high-cholesterol diet and 1 week of alloxan-induced diabetes

| | Diabetic donor n = 5 | Control donor n = 5 | Diabetic recipient n = 10 | Control recipient n = 10 |
|---|---|---|---|---|
| Body weight (kg) | 2.7 ± 0.3 | 3.0 ± 0.3 | 3.0 ± 0.4 | 3.2 ± 0.4 |
| Blood sugar (mmol/L) | 19.7 ± 2.3[a] | 5.9 ± 0.2 | 17.2 ± 3.2[a] | 5.9 ± 0.3 |
| Insulin (U/day) | 4.9 ± 4.2 | — | 4.3 ± 4.3 | — |
| Food intake (g/day) | 139 ± 39 | 130 ± 26 | 140 ± 27 | 130 ± 31 |
| Triglyceride (mmol/L) | 10.8 ± 13.9[a] | 0.8 ± 0.5 | 11.6 ± 11[b] | 1.7 ± 2.4 |
| HDL cholesterol (mmol/L) | 0.3 ± 0.2[a] | 0.8 ± 0.3 | 0.5 ± 0.4[b] | 0.9 ± 0.3 |
| Cholesterol (mmol/L) | 36.9 ± 27.5 | 25.7 ± 10.5 | 52.6 ± 21.9[b] | 33.3 ± 19.4 |
| Free cholesterol (mmol/L) | 13.5 ± 9.0 | 7.1 ± 3.1 | 28.7 ± 18.1[a] | 12.4 ± 11.4 |
| Esterified cholesterol (mmol/L) | 23.5 ± 18.7 | 18.6 ± 7.7 | 23.9 ± 14.5 | 20.2 ± 15.2 |

*Note.* Data are mean ± SD.
[a]$P < 0.01$; [b]$P < 0.05$ different from control rabbits.

diabetic recipient animals and was due to an increase in free cholesterol. Plasma chylomicron apo B48 and apo B100 were significantly higher in the diabetic donor animals compared to control donors (5.2 ± 2.9 and 12.5 ± 4.6 versus 1.4 ± 1.3 and 4.3 ± 4.0 $\mu$g/ml plasma, $P < 0.05$) (Table 2). Plasma chylomicron apo E from diabetic donor rabbits was significantly lower than control animals (0.6 ± 0.2 versus 1.3 ± 0.5 mg/mg apo B, $P < 0.02$). There was a nonsignificant increase in cholesterol triglyceride and phospholipid in diabetic rabbits.

The mean volume of lymph collected in 4 hours was similar for diabetic and control animals (5.5 ± 3.5 versus 5.2 ± 3.0 ml, respectively). The constituents of the lymph chylomicrons (Table 3) used for clearance studies, as mg/h, revealed an almost 5-fold increase in apo B48 ($P < 0.01$) and a more than 3-fold increase in apo B100 ($P < 0.05$) in the diabetic animals. Looking at the lymph chylomicron composition as mg/mg apo B (Table 3), there was less cholesterol and less triglyceride per milligram apo B in the diabetic lymph chylomicron, although in this small number, the differences were not significant. There was a significantly higher apo B48/apo B100 ratio ($P < 0.05$) and significantly less apo E (0.27 ± 0.01 versus 0.62 ± 0.07 mg/mg apo B $P < 0.001$) in the diabetic lymph chylomicron. Thus, the diabetic animals secreted increased numbers of intestinally derived chylomicrons with less lipid and less apo E per particle compared to control animals.

**TABLE 2**
Composition of plasma chylomicrons isolated by ultracentrifugation from donor rabbits at sacrifice

| | Diabetic | Control |
|---|---|---|
| Apo B48 ($\mu$g/ml plasma) | 5.2 ± 2.9[a] | 1.4 ± 1.3 |
| Apo B100 ($\mu$g/ml plasma) | 12.5 ± 4.6[b] | 4.3 ± 4.0 |
| Apo B48/B100 | 0.42 ± 0.2 | 0.32 ± 0.2 |
| Apo E (mg/mg apo B) | 0.6 ± 0.2[b] | 1.3 ± 0.5 |
| Cholesterol (mg/mg apo B) | 105 ± 95 | 84 ± 37 |
| Triglyceride (mg/mg apo B) | 94 ± 142 | 37 ± 10 |
| Phospholipid (mg/mg apo B) | 86 ± 173 | 34 ± 18 |

*Note.* Data are mean ± SD.
[a]$P < 0.05$; [b]$P < 0.02$ different from control rabbits.

**TABLE 3**
Mean composition of injected lymph chylomicrons from donor rabbits isolated by ultracentrifugation 5 hours after gavage with [$^{14}$C]linoleate and [$^{3}$H]cholesterol

| | Diabetic (n = 5) | Control (n = 5) |
|---|---|---|
| Composition (mg/h) | | |
| Cholesterol | 5.1 ± 3.3 | 2.9 ± 1.1 |
| Free cholesterol | 2.1 ± 1.4 | 1.6 ± 0.7 |
| Esterified cholesterol | 3.0 ± 2.5 | 1.4 ± 0.6 |
| Triglyceride | 41.2 ± 13.3 | 30.9 ± 24.5 |
| Phospholipid | 10.7 ± 4.9 | 5.8 ± 4.2 |
| Apo B48 | 0.19 ± 0.10[a] | 0.04 ± 0.02 |
| Apo B100 | 0.22 ± 0.15[b] | 0.07 ± 0.07 |
| Apo E | 0.04 ± 0.03 | 0.03 ± 0.02 |
| Lipid/apo B | 324 ± 285[c] | 2281 ± 1686 |
| Composition (mg/mg apo B) | | |
| Cholesterol | 32 ± 36 | 38 ± 20 |
| Free cholesterol | 8.5 ± 2.4 | 9.1 ± 4.2 |
| Esterified cholesterol | 23 ± 34 | 29 ± 19 |
| Triglyceride | 235 ± 210 | 485 ± 244 |
| Phospholipid | 74 ± 40 | 104 ± 70 |
| Apo E | 0.27 ± 0.01[d] | 0.62 ± 0.07 |
| Apo B48/B100 | 0.97 ± 0.19[b] | 0.62 ± 0.07 |

*Note.* Data are mean ± SD.
[a]$P < 0.01$; [b]$P < 0.05$; [c]$P < 0.02$; [d]$P < 0.001$; different from control rabbits.

**TABLE 4**
Percentage of [$^3$H]cholesterol and [$^{14}$C]linoleate acid cleared from the circulation 5 minutes after injection of donor chylomicrons into recipient animals

| | [$^3$H]cholesterol (%) | [$^{14}$C]linoleate (%) |
|---|---|---|
| Control-control | 84 ± 8 | 88 ± 9 |
| Control-diabetic | 63 ± 6[a] | 75 ± 10[c] |
| Diabetic-control | 67 ± 9[b] | 75 ± 8[c] |
| Diabetic-diabetic | 61 ± 8[a] | 65 ± 10[a] |

*Note.* Data are mean ± SD.
[a]$P < 0.0005$; [b]$P < 0.005$; [c]$P < 0.01$ versus control chylomicron into control recipient.

Chylomicron cholesterol clearance and triglyceride clearance during the first 15 minutes after injection is shown in Figure 1. Five minutes after injection, 84% ± 8% of chylomicron cholesterol had been cleared from the plasma of control rabbits that had been injected with control chylomicrons, compared to 67% ± 9% when they received chylomicrons from diabetic animals ($P < 0.005$) (Table 4). Compared to the control animals that received control chylomicrons, diabetic animals injected with control chylomicrons had cleared 63% ± 6% after 5 minutes ($P < 0.0005$) and 61% ± 8% when they received diabetic chylomicrons ($P < 0.005$). Looking at the disappearance of the

**TABLE 5**
Clearance of chylomicron cholesterol and triglyceride (0- to 15-minute area under the curve)

| | [$^3$H]cholesterol | [$^{14}$C]triglyceride |
|---|---|---|
| Control-control | 426 ± 122 | 347 ± 103 |
| Control-diabetic | 564 ± 106[a] | 511 ± 157[c] |
| Diabetic-control | 589 ± 116[b] | 419 ± 60 |
| Diabetic-diabetic | 648 ± 132[b] | 559 ± 137[b] |

*Note.* Data are mean ± SD.
[a]$P < 0.005$; [b]$P < 0.02$; [c]$P < 0.05$ versus control chylomicron into control recipient.

triglyceride label, control rabbits given control chylomicrons removed 88% ± 9% of the triglyceride by 5 minutes after injection, compared to 75% ± 10% when diabetic chylomicrons were given ($P < 0.01$). Similarly, 75% ± 10% of triglyceride was removed from the plasma of diabetic animals when they received control chylomicrons ($P < 0.01$) and 65% ± 10% when diabetic chylomicrons were given ($P < 0.0005$), compared to control animals injected with control chylomicrons.

Chylomicron cholesterol and triglyceride, expressed as AUC (Table 5), was calculated from the clearance curves for [$^3$H]cholesterol and [$^{14}$C]linoleic acid over the 15-minute period (Figure 2). There was a significant delay in cholesterol

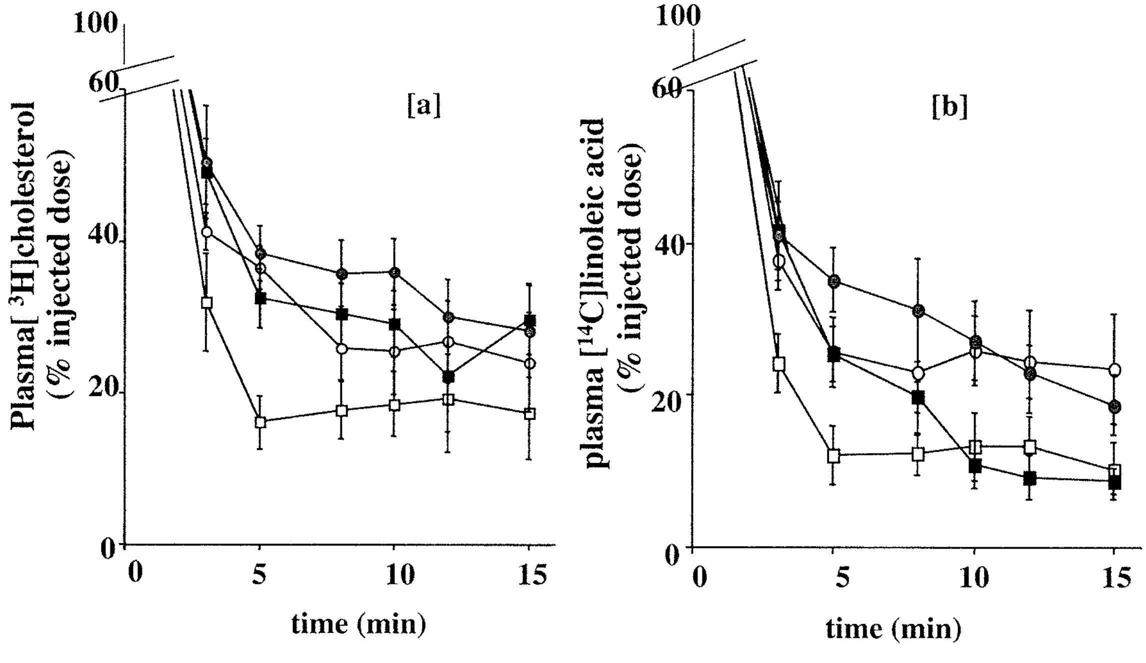

**FIGURE 2**
Chylomicrons isolated from lymph of diabetic and control donor rabbits 4 hours following an oral load of [$^3$H]cholesterol and [$^{14}$C]linoleic acid was reinjected into 5 diabetic and 5 control recipient rabbits. (*a*) Clearance of [$^3$H]cholesterol and (*b*) clearance of [$^{14}$C]linoleic acid. Control donor into control recipient (*white squares*), diabetic donor into control recipient (*black squares*), control donor into diabetic recipient (*white circles*), and diabetic donor into diabetic recipient (*black circles*). Data are mean ± SE.

clearance when chylomicrons from control animals were injected into the diabetic recipient, compared to the control chylomicron into the control recipient ($P < 0.005$). There was a very similar delay in AUC for cholesterol clearance when the diabetic chylomicron was injected into the control recipient ($P < 0.02$) and when chylomicrons from the diabetic donor were injected into the diabetic recipient ($P < 0.02$, different from control to control). The pattern for triglyceride clearance was somewhat different. Triglyceride clearance was significantly delayed when control chylomicrons were injected into the diabetic recipient ($P < 0.05$), compared to control recipient. Injecting chylomicrons from the diabetic donor into the diabetic recipient resulted in a delay of triglyceride clearance slightly greater than that found when the control particles were injected into the diabetic recipient ($P < 0.02$).

We examined whether the lymph chylomicron apo B or lipid/apo B composition could have influenced the clearance of the particle. However, we were unable to find any relationship between these parameters and either cholesterol or triglyceride clearance. There were positive correlations between AUC for triglyceride removal and AUC for cholesterol clearance ($r = .59$, $P < 0.01$) and also AUC for triglyceride removal and blood glucose on day of experiment ($r = .57$, $P < 0.01$).

### DISCUSSION

The cholesterol-fed rabbit is a widely used model for experimental atherosclerosis research [16]. We therefore fed our animals a 0.5% cholesterol diet, because this diet is known to produce atherosclerosis in rabbits [20]. Having decided on our atherosclerotic model, we made some of the animals diabetic using alloxan; our hypothesis being that alterations in chylomicron composition might be partly responsible for the delay in chylomicron clearance in diabetes. Examination of chylomicrons isolated from intestinal lymph in the postprandial state ensures pure intestinally derived particles, uncontaminated by hepatically derived particles. In rabbits, this lymph contains both apo B48 and apo B100, because the rabbit has less complete editing of intestinal apo B100 mRNA to apo B48 mRNA than human [21]. In the present study, lymph chylomicron particles from diabetic rabbits contained significantly more apo B48 and apo B100 than particles from control animals, and the triglyceride/apo B48 ratio was significantly reduced in the diabetic animals. Thus, the major effect of diabetes on the chylomicron was the production of a larger number of smaller lipid-deficient particles. The ratio of apo B48/apo B100 in plasma was very different, demonstrating the considerable amount of large hepatically derived very-low-density lipoprotein (VLDL) found in the chylomicron fraction of plasma.

Apo E plays an important regulatory role in chylomicron and VLDL metabolism through its recognition by the LDL receptor, the LSR, LRP, and glycosaminoglycans [11–13]. A reduction in apo E on the particle or alteration in the apo E genotype (apo E2) has been shown to reduce clearance of the particle [22, 23]. Our results show a reduction in apo E on both lymph and plasma chylomicron particles in the diabetic rabbit, demonstrating that the apo E deficiency in lymph is not corrected in the plasma. Lenich and colleagues [14], some years ago, demonstrated that insulin deficiency in the cholesterol-fed rabbit reduced apo E mRNA in the liver and adrenals. The intestine, however, was not studied. In the diabetic rat, Levy and coworkers [9] showed that there was significantly less lymph apo E and apo Al secreted per hour and more apo B than in control animals. They did not report particle clearance. Feingold and colleagues [10], also using the rat, showed no difference in clearance of the diabetic chylomicron particle when injected into normal rats, but there was no information on apo E. In human studies, Syvanne and coworkers [24], in a preliminary report, demonstrated enrichment of apo E on postprandial triglyceride-rich lipoproteins in diabetic patients. The [$^3$H]cholesterol label in the chylomicron particle from control rabbits in our study, when injected into the diabetic animal, resulted in a significantly increased retention time, demonstrating a defect in clearance of the particle. No correction was made for the increased plasma volume in the diabetic animal, but if we had used a slightly higher value in the hyperglycemic animals to correct for the higher osmotic pressure of the glucose, the results would have demonstrated an even further delay in clearance. It is possible that the delayed clearance of the chylomicron particle when injected into the diabetic recipient was in part due to their increased pool size of triglyceride-rich lipoproteins. However, clearance of the [$^3$H]cholesterol label was also impaired when the diabetic chylomicron was injected into the control animal, whereas clearance of [$^{14}$C]linoleic acid was normal. This suggests that, even with normal hydrolysis of triglyceride, structural abnormalities prevented uptake of the diabetic chylomicron through its many pathways. Because there was no difference in the chylomicron triglyceride/apo B between the 2 groups, we used a constant amount of chylomicron triglyceride when injecting the chylomicron particle into the recipient animals. Delayed clearance of cholesterol in the diabetic recipient animals may in part have been due to the increased particle load endogenously present in the diabetic recipient animal. Redgrave and colleagues [25] have shown that particle number plays a more important role than particle size in determining the rate of chylomicron clearance. Furthermore, insulin has been shown to upregulate the B/E receptor [26] and thus the diabetic state of relative hypoinsulinemia may impede clearance of the chylomicron through downregulation of the receptor. Delayed clearance of the diabetic particles when injected into the control animals strongly suggests a compositional abnormality of the particle.

In our study, the deficiency in apo E, which is the major ligand for the hepatic receptors, would seem to be the main reason for the defective clearance of diabetic chylomicrons when injected into control animals. The reason why apo E should be deficient in particles from the diabetic animals may be related to the effect of insulin as a regulator of apo E [14] or due to an inability of apo E to bind to the diabetic particle. It has been suggested that there is excess of apo E in the serum and that apo E concentrations are usually well above the minimum level necessary for normal lipoprotein clearance [22]. The fact that we found apo E deficiency in both the lymph chylomicron and in the serum chylomicron fraction favors a defect in binding of apo E to the particle as the explanation.

Examination of turnover of the [$^{14}$C] label, a marker of triglyceride clearance, demonstrated the expected delay when the diabetic lymph chylomicron was injected into the diabetic animal. We presume this was due to a defect in lipoprotein lipase, which is thought to be a major reason for the hypertriglyceridemia of diabetes [27]. It would be expected that an excess of triglyceride-rich lipoproteins in the circulation would delay clearance of injected triglyceride-rich particles due to competition for receptors [28] and of course we had a 5-fold increase in plasma triglyceride in the diabetic recipient animals. We found a positive correlation between blood sugar on the day of experiment and triglyceride retention time, again suggesting a relationship with insulin deficiency. The control animal was able to clear triglyceride from the chylomicron particle produced by the diabetic animal over a 15-minute period, but the initial clearance over the first few minutes was significantly delayed. Levy and coworkers [9] showed convincingly that there was delayed clearance of chylomicron triglyceride from diabetic donor rats when injected into nondiabetic animals in the first few minutes after injection, but they did not study cholesterol clearance. In our study, it is interesting that by 15 minutes, the initial delay in triglyceride clearance had been overcome.

This study demonstrates that in the cholesterol-fed rabbit, diabetes results in an increase in the number of chylomicron particles, which are apo E depleted. Both diabetic and control animals had delayed clearance of diabetic chylomicron cholesterol, suggesting mechanisms to explain the increased atherogenesis in diabetes, because chylomicron remnant particles have been shown to be particularly atherogenic.

### REFERENCES

[1] Tomkin, G. H., and Owens, D. (2001) ApoB lipoproteins, diabetes and atherosclerosis. *Diabetes Metab. Rev.,* **17,** 27–43.

[2] Takeichi, S., Yukawa, N., Nakajima, Y., Osawa, M., Saito, T., Seto, Y., Nakano, T., Saniabadi, A. R., Adachi, M., Wang, T., and Nakajima, K. (1999) Association of plasma triglyceride-rich lipoprotein remnants with coronary atherosclerosis in cases of sudden cardiac death. *Atherosclerosis,* **142,** 309–315.

[3] Shaw, H. E., Hodge, A. M., deCourten, M., Chitson, P., and Zimmet, P. Z. (1999) Isolated postchallenge hyperglycaemic confirmed as a risk factor for mortality. *Diabetologia,* **42,** 1050–1054.

[4] Anderson, J. H., Brunelle, R. L., Koivisto, V. A., Pfutzner, A., Trautmann, M. E., Vignati, L., DiMarchi, R., and the Multicenter Insulin Lispro Study Group (1997) Reduction of postprandial hyperglycaemia and frequency of hypoglycaemia in IDDM patients on insulin-analog treatment. *Diabetes,* **46,** 265–270.

[5] Curtin, A., Deegan, P., Owens, D., Collins, P., Johnson, A., and Tomkin, G. H. (1994) Alterations in apolipoprotein B-48 in the post-prandial state. *Diabetologia,* **37,** 1259–1262.

[6] Curtin, A., Lenihan, K., Deegan, P., Owens, D., Johnson, A., Collins, P., and Tomkin, G. H. (1995) Intestinally derived lipoprotein particles in non-insulin-dependent diabetic patients with and without hypertriglyceridaemia. *Acta Diabetologica,* **32,** 244–250.

[7] Curtin, A., Deegan, P., Owens, D., Johnson, A., Collins, P., and Tomkin, G. H. (1996) Elevated triglyceride-rich lipoproteins in diabetes: A study of apo B48. *Acta Diabetologica,* **33,** 205–210.

[8] Taggart, C., Gibney, J., Owens, D., Collins, P., Johnson, A., and Tomkin, G. H. (1997) The role of dietary cholesterol in the regulation of post-prandial apolipoprotein B48 levels in diabetes. *Diabetic Medicine,* **14,** 1051–1058.

[9] Levy, E., Shafrif, E., Ziv, E., and Bar-On, H. (1983) Composition, removal and metabolic fate of chylomicrons derived from diabetic rats. *Biochim. Biophys. Acta,* **834,** 376–385.

[10] Feingold, K. R., Lear, S. R., and Felts, J. M. (1986) The disappearance from the circulation of chylomicrons obtained from control and diabetic rats. *Endocrinology,* **121,** 475–480.

[11] Weisgraber, K. H. (1994) Apoprotein E: Structure-function relationships. *Adv. Protein Chem.,* **45,** 249–302.

[12] Yen, F. T., Mann, C. J., Guermani, L. M., Hannouche, N. F., Hubert, N., and Hornick, C. A. (1994) Identification of a lipolysis-stimulated receptor that is distinct from the LDL receptor and the LDL receptor-related protein. *Biochemistry,* **33,** 1172–1180.

[13] Mahley, R. W., and Ji, Z.-S. (1999) Remnant lipoprotein metabolism: Key pathway involving cell surface heparin sulphate proteoglycans and apolipoprotein E. *J. Lipid Res.,* **40,** 1–16.

[14] Lenich, C. M., Chobanian, A. V., Brecher, P., and Zannis, V. I. (1991) Effect of dietary cholesterol and alloxan-diabetes on tissue cholesterol and apolipoprotein E mRNA levels in the rabbit. *J. Lipid Res.,* **32,** 431–438.

[15] Yamamoto, K., Shimano, H., Shimada, M., Kawamura, M., Gotoda, T., Harada, K., Ohsuga, Ji., Yazaki, Y., and Yamada, N. (1995) Overexpression of apolipoprotein E prevents development of diabetic hyperlipidaemia in transgenic mice. *Diabetes,* **44,** 580–585.

[16] Finking, G., and Hanke, H. (1997) Nickolaj Nikolajewitisch Anitschkow (1885–1964) established the cholesterol-fed rabbit as a model for atherosclerosis research. *Atherosclerosis,* **135,** 1–7.

[17] Markwell, M. A. K., Haas, S. M., Bieber, L. L., and Tolbert, N. E. (1978) A modification of the Lowry procedure to simplify protein determination in membrane and lipoprotein samples. *Anal. Biochem.,* **87,** 206–210.

[18] Phillips, C., Murugasu, G., Owens, D., Collins, P., Johnson, A., and Tomkin, G. H. (2000) Improved metabolic control reduces the number of postprandial apolipoprotein B48-containing particles in Type 2 diabetes. *Atherosclerosis,* **148,** 283–291.

[19] Kotite, L., Bergeron, N., and Havel, R. J. (1995) Quantitation of apolipoproteins B100, B48 and E in human triglyceride-rich lipoproteins. *J. Lipid Res.,* **36,** 890–900.

[20] Bocan, T. M., Mueller, S. B., Mazur, M. J., Uhlendorf, P. D., Brown, E. Q., and Kieft, K. A. (1993) The relationship between the degree of dietary induced hypercholesterolaemia in the rabbit and atherosclerotic lesion formation. *Atherosclerosis,* **102,** 9–22.

[21] Greeve, J., Altkemper, I., Dieterich, J.-H., Greten, H., and Windler, E. (1993) Apolipoprotein B editing in 12 different mammalian species: Hepatic expression is reflected in low concentrations of apo B-containing plasma lipoproteins. *J. Lipid Res.,* **34,** 1367–1376.

[22] Hasty, A. H., Linton, M. R. F, Swift, L. L., and Fazio, S. (1999) Determination of the lower threshold of apolipoprotein E resulting in remnant lipoprotein clearance. *J. Lipid Res.,* **40,** 1529–1538.

[23] Weisgraber, K. H., Innerarity, T. L., and Maley, R. W. (1982) Abnormal lipoprotein receptor binding activity of human E apoprotein due to cysteine-arginine interchange at a single site. *J. Biol. Chem.,* **257,** 2818–2821.

[24] Syvanne, M., Rosseneu, M., Labeur, C., Hilden, H., and Taskinen, M. R. (1994) Enrichment with apo E characterises postprandial TG-rich lipoproteins in patients with non-insulin-dependent diabetes mellitus and coronary artery disease; a preliminary report. *Atherosclerosis,* **105,** 25–34.

[25] Redgrave Mamo, J. C. L., Hirano, T., Sainsbury, A., Fitzgerald, A. K., and Redgrave, T. G. (1992) Hypertriglyceridaemia is exacerbated by slow lipolysis of triglycerol-rich lipoproteins in fed but not fasted streptozotocin diabetic rats. *Biochim. Biophys. Acta,* **1128,** 132–138.

[26] Chait, A., Beirman, E. L., and Albers, J. J. (1997) Low density lipoprotein receptor activity in cultured skin fibroblasts: Mechanisms of insulin induced stimulation. *J. Clin. Invest.,* **64,** 1309–1319.

[27] Eckel, R. H. (1989) Lipoprotein lipase: A multifunctional enzyme relevant to common metabolic diseases. *N. Engl. J. Med.,* **320,** 1060–1068.

[28] Demacker, P. N. M., Bredie, S., Vogelaar, J. M., Hectors, M., van Heijst, P., Stuyt, P., and Stalenhoef, A. (1981) $\beta$-VLDL accumulation in familial dysbetalipoproteinaemia is associated with increased exchange or diffusion of chylomicron lipids to apo B100 containing triglyceride-rich lipoproteins. *Atherosclerosis,* **38,** 301–312.

